# Randomized Crossover Study of Auricular Plaster Therapy to Relieve Dental Anxiety in Children

**DOI:** 10.3389/fpsyt.2022.862575

**Published:** 2022-05-31

**Authors:** Jiangtao Wang, Jie Zhang, Dalei Sun

**Affiliations:** ^1^Department of Psychiatry, Xi'an Union Hospital, Xi'an, China; ^2^Psychological Medicine Center, The First Affiliated Hospital of Xinjiang Medical University, Ürümqi, Xinjiang, China; ^3^Central Laboratory, School of Public Health, Xinjiang Medical University, Ürümqi, China; ^4^Stomatology Center, The Affiliated Hospital of Hangzhou Normal University, Hangzhou, China; ^5^Departments of Pediatric Dentistry, Oral Health, and Preventive Dentistry, The First Affiliated Hospital of Xinjiang Medical University (Affiliated Stomatology Hospital), Research Institute of Stomatology, Xinjiang Uygur Autonomous Region, Ürümqi, China

**Keywords:** child dental anxiety, auricular-plaster therapy, trait anxiety, non-trait anxiety, acupressure

## Abstract

**Objective:**

To determine if auricular plaster therapy (APT) can alleviate dental anxiety in children aged 9 or 10 years old.

**Methods:**

A crossover research was conducted on children with at least two deep-arrested deciduous molar caries (*N* = 80?). The first group (*N* = 40) received APT intended to reduce anxiety prior to the first caries treatment, whereas the second group (*N* = 40) received placebo/control APT (no anticipated impact on anxiety). The APT approaches were exchanged after a washout period following the initial caries treatment. Additionally, both groups were also informed and given a demonstration regarding the procedures and equipment prior to their use as part of a Tell-Show-Do (TSD) protocol. The dentists, children, and parents were all involved in assessing the level of anxiety using general anxiety scales. Moreover, the average heart rate and salivary cortisol concentration, both of which are indications of anxiety, were compared between the pre- and post-intervention periods. The participants were unaware of the type of APT that was employed (anti-anxiety vs. control). To avoid inadvertently influencing the outcome, all psychologists, investigators, and data recorders were blinded to the randomized subject sequence.

**Results:**

Children treated with anti-anxiety APT demonstrated significantly higher levels of obedience than children treated with control APT (*P* < 0.05). In addition, children treated with APT had a lower average heart rate while awaiting treatment, undergoing local anesthesia, and receiving dental caries treatment (*P* < 0.05). These children had reduced salivary cortisol levels while awaiting treatment (*P* < 0.05).

**Conclusion:**

Anti-anxiety APT can help relieve dental anxiety in children.

## Introduction

Child dental anxiety (CDA) is a negative mental condition that arises in children who require dental treatment (1). This anxiety may manifest in various behaviors such as muscle tensing, fist-clenching and limb-twisting, frequent urination, and nausea (2). These behaviors impede the children's access to treatment and may lead to incomplete or postponed treatment (1). Between 1971 and 2015, a systematic, retrospective study revealed that ~10% of children and adolescents suffer from dental anxiety (3). However, other studies have suggested that the rate may be significantly higher (4, 5), and survey data in several regions of China indicate that CDA prevalence may be as high as 50% (6–8). Rather than easing CDA, modern behavior management techniques focus on ensuring children are obedient. Furthermore, several behavior management methods may have a psychological impact on children and parents, resulting in severe CDA.

Acupressure is the application of pressure to conventional acupuncture points that have been evaluated for various indications ranging from cancer to obesity (9). A recent report suggested that acupressure can directly alleviate anticipatory anxiety in adults (10). Auricular acupressure, as well as conventional acupuncture, has been shown to facilitate anesthesia and analgesia and relieve anxiety (11, 12), prompting subsequent investigations into its potential benefits for anxiety. Additionally, point acupuncture can help reduce pain and autonomic excitability when children undergo local dental anesthesia (40). Previously, we have established that auricular acupressure can help nail biters overcome their anxiety and break the habit (13). Thus, these data indicated that auricular acupressure may help alleviate anxiety and pain in children receiving dental caries therapy. Auricular plaster treatment (APT) is a technique that utilizes magnetic “seeds” to apply pressure to specific areas of the ear. Whether the APT can alleviate CDA is our purpose of designing this study.

## Methods

### Sample Size Calculation

The sample size calculation was performed for a comparison between APT and control APT with a hypothesis of superiority and a two-side (α = 0.05) paired-samples-test to provide 80% power. Expecting to find a 30% difference in anxiety levels between the Method A and Method B, the number of participants needed was calculated to be 19 for each group to complete the study (13, 40). However, given the high trial dropout rates for a two-sequence, two-period crossover design, we expected that recruitment of more than 40 enrollees for each group would yield the above number of completers.

### Recruitment

Eighty children treated at pediatric dentistry department (The First Affiliated Hospital of XinJiang Medical University, Ürümqi, China) between March 2019 and October 2019 were selected for the study, which was approved by our institution's institutional review board (Ethics approval No. K202012-09). The inclusion criteria were: (i) First-visit Han Chinese children aged 9 or 10 years old, with two or more deep-arrested deciduous molar caries that have not invaded the dental pulp, (ii) the children were with their parents, who could communicate with the researchers in Mandarin. The exclusion criteria were: (i) the presence of temporomandibular joint disorders, (ii) the need for emergency oral treatment, (iii) children who were receiving acupuncture and moxibustion therapy, (iv) children with diseases or deformities affecting the external auditory meatus, (v) children who had taken analgesics, (vi) children with psychiatric disorders, (vii) children with blood coagulation disorders, (viii) children with disorders of mental development, (ix) children who refused to participate, and (x) children whose guardians refused to participate in the study. All children and their guardians provided consent for participation in the study and publication of their data.

#### Caries Treatment

The patients' caries treatments were divided into three consecutive stages: waiting for the treatment, local anesthesia, and dental caries treatment. The waiting stage (~10 min) was the time after the children had entered the consulting room but before local anesthesia was performed. Local anesthesia refers to the time during which disinfection and local anesthesia were performed. The caries removal stage refers to the time during which deep dental caries was removed by turbine dental handpiece. There were two separate treatments evaluated in this study, with one tooth treated each session, and the whole treatment process was completed by the same dentist.

#### Trial Sequence

**Figure 1:**
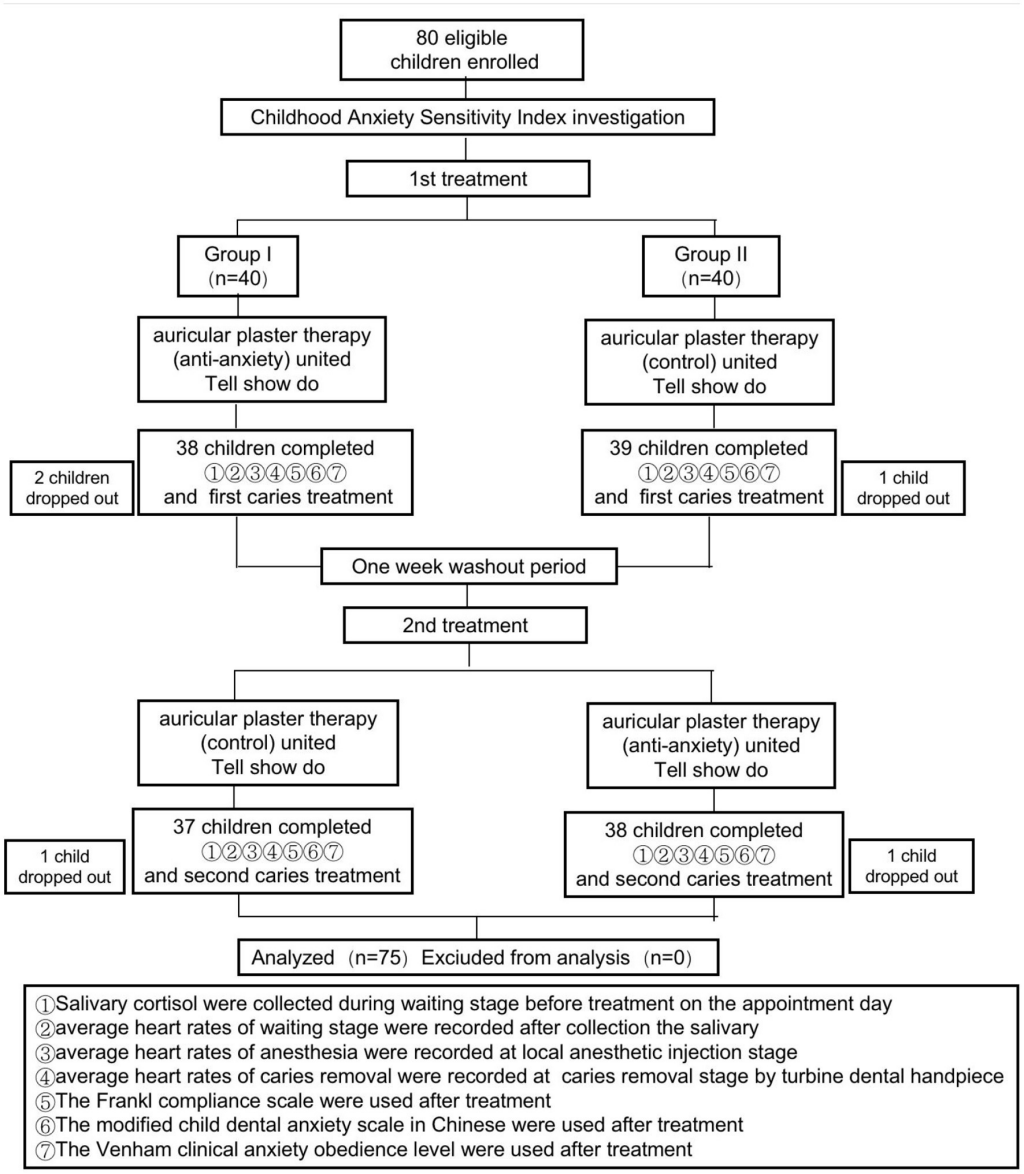
A flowchart showing the treatment schedule and patient retention.

**Figure 2:**
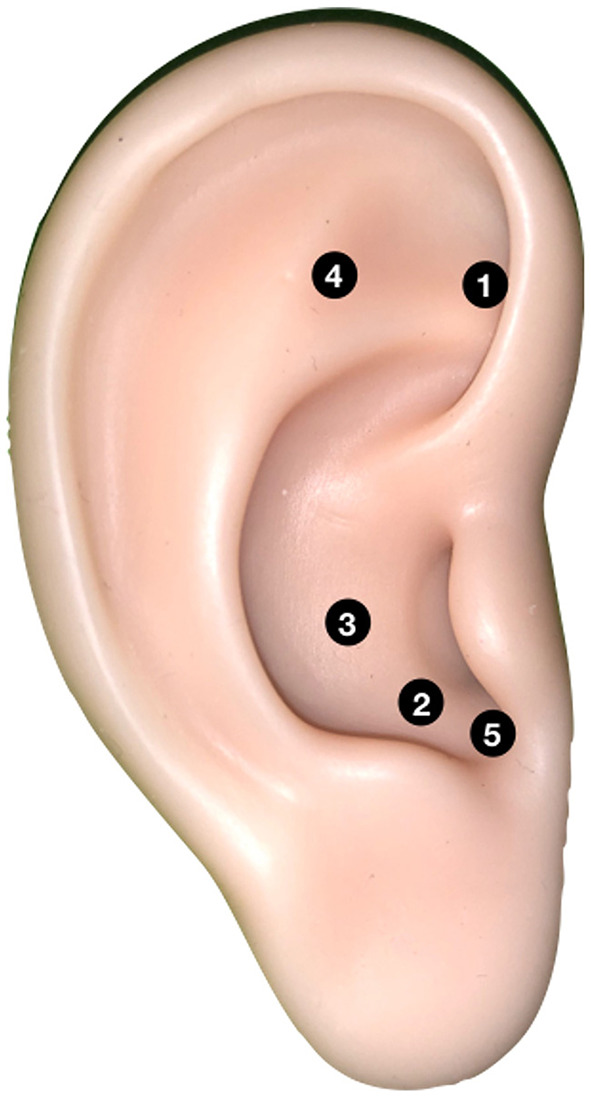
The anxiety-reducing auricular acupressure points are used for APT. 1. Sympathetic point (MA-AH7), 2. Sanjiao point (MA-IC4), 3. Heart point (MA-IC), 4. Shenmen point (MA-TF1), and 5. Adrenal gland point (MA-TG).

**Figure 3:**
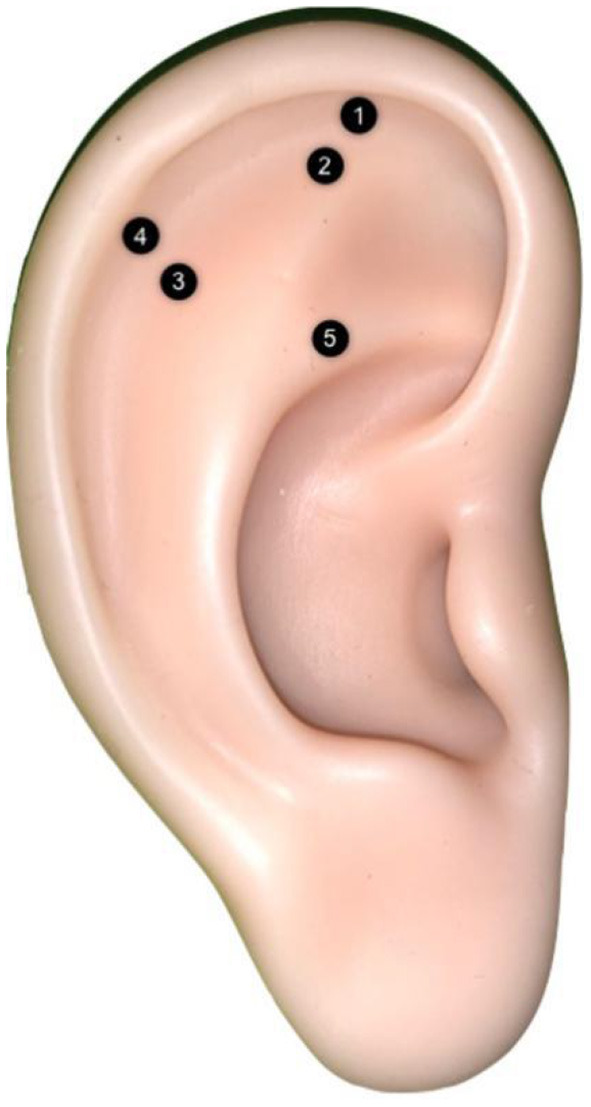
The control (non-anxiety-reducing) auricular acupressure points are used for APT. 1. Heel point (MA-AH1), 2. Ankle point (MA-AH2), 3. Knee point (MA-AH3), 4. Hip point (MA-AH4), and 5. Buttock point (MA-AH5).

All children and their parents provided verbal and written consent to participate in the study and to the proposed treatments. The trial consisted of two parallel procedures and lasted from March to October 2019 (Figure 1). One procedure comprised anxiety-reducing auricular acupressure plus Tell-Show-Do (TSD). APT was performed using five pressure points (Figure 2) with demonstrated anxiety-reducing effects: the sympathetic point (MA-AH7), Sanjiao point (MA-IC4), heart point (MA-IC), Shenmen point (MA-TF1), and adrenal gland point (MA-TG) (13–16). The other method comprised “placebo”/control auricular acupressure plus TSD. The “placebo” APT was performed at five points not considered to have anxiety-reducing effects: the heel point (MA-AH1), ankle point (MA-AH2), knee point (MA-AH3), hip point (MA-AH4), and buttock point (MA-AH5) (Figure 3). The TSD procedure was the same for both APT methods.

#### Study Randomization and Subject Grouping

Since this was a crossover study, all children were treated using both methods (one method for the first caries treatment, the other for the second). The sequence of the procedures was randomized using a random number table in Excel, with half of the children being randomized to each group. The randomization was performed by an individual who was not directly involved in the clinical aspects of the research. Individuals assigned odd numbers were allocated to Group 1 (anti-anxiety APT first) and those with even numbers were allocated to Group 2 (control APT first). If the allocation (even/odd numbers) was uneven, the group with an excess number was re-randomized until the two groups were even. The allocation for each child was placed in an opaque sealed envelope that was opened independently by the acupressurist immediately before the first treatment to determine the placement of the auricular plasters.

The participants were blinded to the type of APT used (anti-anxiety vs. control). The psychologists, investigators, and data recorders were also blinded to the sequence into which the participants were randomized to prevent them from inadvertently influencing the outcome.

#### Tell-Show-Do

As noted, a TSD procedure was used for all patients at each treatment during this study, regardless of the randomization. For the TSD procedure, the researchers communicated genially with the children in plain language (Tell) to explain the objective of dental caries treatment and the therapy to be adopted and to tell them how to be obedient during the treatment. The researchers then had the children watch a video about dental caries treatment (Show) so that they could understand the process of treatment and the hazards of failing to receive timely treatment. Meanwhile, the relevant medical equipment, including the high-speed turbine dental handpiece, three-purpose gun, mouth mirror, excavator, and probe, were shown to the children. Finally, the researchers let the children directly touch all the medical equipment, and encouraged them to put the equipment in their mouths to feel it (Do).

#### Auricular Acupressure

**Figure 4:**
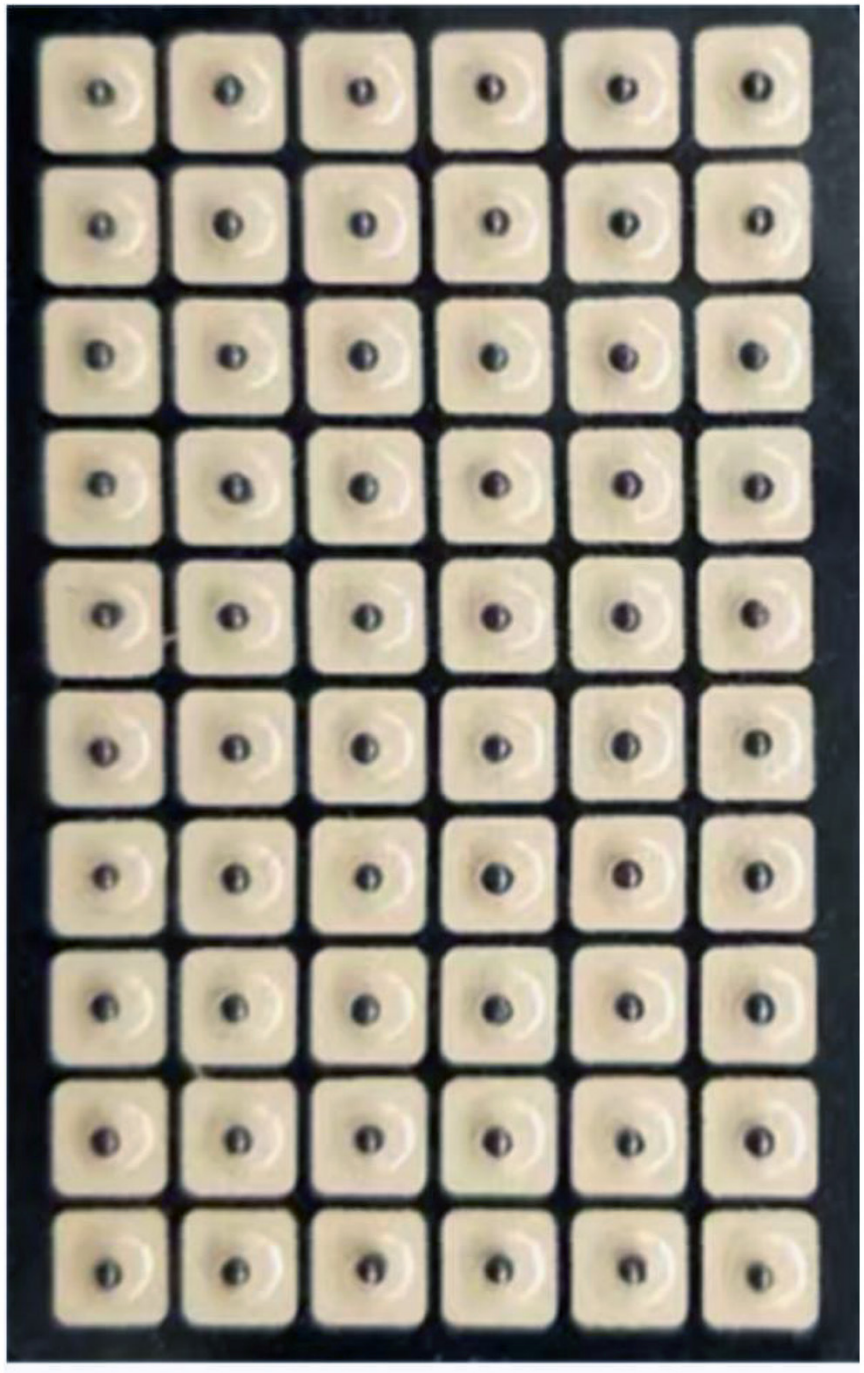
The adhesive-backed ear patch “seeds” used for APT.

Magnetic “seeds” (Suzhou Gusu Acupuncture & Moxibustion Appliance Co., Ltd.; Figure 4), which had an adhesive backing, were applied by an acupressurist with more than 10 years of experience. The acupressurist stuck these onto one of each child's ears based on the randomization. After 7 days, these were removed, and five new seeds were stuck on the same points of the other ear. The participants pressed the acupressure points with the seeds three times per day for 20 s under parental supervision as recommended in a previous study (17). The children's self-compression led to feelings of local acid/a sour taste, mild swelling, pain, and/or a burning sensation. None of the patients stopped the treatment due to these sensations.

#### Salivary Cortisol (SCO) Determination

Saliva samples were collected for analysis of the cortisol level by passive drooling (18, 19). To minimize the impact of the circadian rhythm, all saliva samples were collected at 15:30–16:00 during waiting stage before treatment 1 and treatment 2 on the appointment day. All samples were taken during the waiting stage. The children were prohibited from eating or drinking for 2 h before sampling. Prior to saliva collection, they gargled with clear water and disgorged the water completely, then sat on a general oral treatment chair and did not speak for 5 min before sampling. For saliva collection, the child was asked to first swallow the saliva present in their mouth, and then to slowly lean the upper part of the body forward with their head bent slightly until they salivated naturally, with saliva flowing into the sterile collection tube. They were instructed not to swallow saliva or move their tongue or lips during collection. At least 3 ml of saliva was collected from each child while they remained in this position. The collected saliva samples were centrifuged at 3,000 r/min for 20 min. The resulting clear supernatant was collected, put into a sterile Eppendorf tube, and stored at −80°C until analysis. An ELISA kit (DRG SCO ELISA; DRG International, USA) was used to determine the level of SCO as specified by the manufacturer.

#### Heart Rate Measurement

Anxiety typically leads to an elevated heart rate. Thus, a child portable electrocardiogram monitor was used to measure each child's heart rate during the three stages of treatment 1 and treatment 2. We record the average heart rate at waiting stage (after collection the salivary), at anesthesia stage (local anesthetic injection stage), and at the Caries removal stage by turbine dental handpiece. Statistical analysis was conducted on the average heart rates for the three stages.

#### The Childhood Anxiety Sensitivity Index (CASI)

The Chinese version of the CASI (CASI-C) was adopted to assess trait anxiety (20). After the 80 children were enrolled in the study, a child psychiatrist guided children to complete the questionnaire to ensure that the children can correctly understand and choose all the questions, which are in line with their status. The cumulative scores were the total points for anxiety (0–36 points). There were 18 items included in the questionnaire. Three-grade scoring criteria were adopted (where 0 represents never; 1 represents sometimes; 2 represents often). The higher the score, the higher the children's anxiety level. A score of 9 was taken as the cutoff score for boys, while 11 was taken as the cutoff score for girls, and scores equal to or above the cut-off indicated that the child was suffering trait anxiety.

#### Evaluation of Dental Anxiety Using Different Scoring Systems

The Frankl compliance scale (FCS) was used by the researchers to evaluate the children's behaviors throughout the treatment (21, 22). After making a double-blind evaluation, the two researchers (Dr. Wang zhiheng and Dr. Zhu shaojun) took the average of their results and set a score range of 1–4. The higher the score, the better the obedience.

The modified child dental anxiety scale in Chinese (MCDAS) was adopted as a self-assessment for the children (23). Two pediatric dentistry postgraduates, who underwent normalization training about how to properly utilize the scale, provided instructions to the children and answered any questions that were raised. The survey was conducted in the waiting room. The children filled in the questionnaire under the guidance postgraduatests (Sun xinixn and Wang bangyao). The parents participated in the scoring process, and the score range was set to 8–40. The higher the score, the more severe the CDA.

The Venham's clinical anxiety obedience level rating scale was adopted to allow parents to assess their children's anxiety (24, 41). After providing the parents with a detailed introduction to the scale and having them watch a video about the children's obedience level, a dental specialist (Dr. Li boqi) participated in the scoring process to answer the parents doubt. We take the average score after parent's rating, and higher scores indicated more severe CDA and poorer obedience.

#### Statistical Analysis

The test data were statistically analyzed using SPSS17.0. Differences related to gender were compared using Fisher's exact test, and the two groups were compared in terms of age using completely randomized *t*-testing. The two methods (anti-anxiety and control acupressure) were compared by the paired *t*-test. The results are expressed as the means ± standard deviation, and the test standard α was 0.05.

## Results

### Subjects

A total of 80 children who met the inclusion criteria were randomly divided into two groups of 40 children. Two children in Group 1 and one in Group 2 were lost by the end of the first treatment. There was a 1-week washout interval between the first and second treatments. One child in each group was lost during the wash-out period. Therefore, 75 children underwent both treatments, 37 in Group 1 and 38 in Group 2, and these were included in the statistical analysis (Figure 1).

### Comparison of Basic Information Between the Two Groups

The groups were gender-balanced, in which female children accounted for 45.9% of those in Group 1 and 47.4% in Group 2, (X^2^ = 0.015), and the difference was statistically insignificant (*P* > 0.05).

### Comparison of the Various Dental Anxiety Scores

As shown in Table 1, children treated with the anti-anxiety auricular-plaster received a mean score of 3.33 ± 0.50 on the dentist-scored Frankl assessment, whereas the control group had a mean score of 3.13 ± 0.62 (*t* = 2.632, *P* < 0.05). In the parent-scored Venham clinical anxiety assessment, the children treated with the anti-anxiety method received a mean score of 0.99 ± 0.73, while those treated with the control method had a mean score of 1.17 ± 0.89, which was a statistically significant difference (*t* = 3.951, *P* < 0.05). In the child-scored MCDAS, those treated using the anti-anxiety approach had a mean score of 16.51 ± 5.29, while those treated using the control auricular-plaster had a mean score of 17.60 ± 6.89 (*t* = 2.259, *P* < 0.05).

**Table 1:** Comparison of TSD with and without APT.

	**TSD + APT**	**TSD + control**	***t***	***P***
**Heart rate**				
Waiting	83.25 ± 8.68	86.85 ± 8.81	3.554	0.001*
Anesthesia	90.00 ± 10.12	91.75 ± 10.43	3.422	0.001*
Caries removal	88.84 ± 9.83	90.59 ± 10.02	3.391	0.001*
**CDA score**				
Dentist-Frankel	3.33 ± 0.50	3.13 ± 0.62	2.632	0.010*
Parents-Venham	0.99 ± 0.73	1.17 ± 0.89	3.951	0.000*
Children-MCDAS	16.51 ± 5.29	17.60 ± 6.89	2.259	0.027*
SCO concentration	7.58 ± 2.30	8.01 ± 2.38	4.333	0.000*

*APT, anti-anxiety auricular-plaster therapy; CDA, child dental anxiety; Control, auricular-plaster therapy without anti-anxiety effects; MCDAS, modified child dental anxiety scale in Chinese; SCO, salivary cortisol; TSD, tell-show-do*.

*p < 0.05 for comparisons of TSD + APT versus TSD + control.

### Differences in the Salivary Cortisol Levels

In a comparison of the SCO concentrations recorded at waiting stage, the children treated with the anti-anxiety approach had a mean value of 7.58 ± 2.30 while those treated with the control approach had a mean value of 8.01 ± 2.38, which was a statistically significant difference (*t* = 4.333, *P* < 0.05; Table 1).

### Differences in the Heart Rates

Additionally, there were substantial variations in heart rates between the APT and control groups (Table 1). As the children awaited the treatment, those treated using the anti-anxiety method had a heart rate of 83.25 ± 8.68 beats/min while those treated with the control had a mean heart rate of 86.85 ± 8.81 beats/min, which was a statistically significant difference (*t* = 3.554, *P* < 0.05). During local anesthesia, the mean heart rate was 90.00 ± 10.12 beats/min in those using the anti-anxiety method, while it was 91.75 ± 10.43 beats/min in those using the control method (*t* = 3.422, *P* < 0.05). During caries removal stage, the heart rate was 88.84 ± 9.83 beats/min in those using the anti-anxiety method, and 90.59 ± 10.02 beats/min in those treated using the control method (*t* = 3.391, *P* < 0.05).

### Comparison of Children With and Without Trait Anxiety

Since we considered that children with pre-existing trait anxiety were more likely to benefit from anxiety-reduction treatments (25), we compared outcomes according to whether the children had trait anxiety. We divided the 75 children into two subgroups using the questionnaire responses of 21 children with trait anxiety and 54 children with non-trait anxiety and then analyzed the two subgroups separately for the effects of the auricular plasters (Table 2).

**Table 2:** Comparison of anxious and non-anxious children.

		**TSD + APT**	**TSD + control**	***t***	***p***
Trait anxiety	**Heart rate**				
	Waiting	81.67 ± 9.93	90.38 ± 9.89	3.381	0.003*
	Anesthesia	90.52 ± 11.92	94.00 ± 11.43	4.645	0.000*
	Caries removal	87.62 ± 11.04	91.29 ± 10.77	4.485	0.000*
	**CDA score**				
	Dentist-Frankel	3.17 ± 0.46	2.71 ± 0.49	2.939	0.000*
	Parents-Venham	1.67 ± 0.70	2.14 ± 0.79	5.423	0.000*
	Children-MCDAS	20.57 ± 5.86	25.52 ± 6.59	4.308	0.000*
	SCO concentration	7.96 ± 1.95	9.09 ± 1.91	6.518	0.000*
Non-trait anxiety	**Heart rate**				
	Waiting	83.87 ± 8.16	85.48 ± 8.04	1.862	0.068
	Anesthesia	89.80 ± 9.45	90.87 ± 9.99	1.714	0.092
	Caries removal	89.31 ± 9.38	90.31 ± 9.81	1.625	0.110
	**CDA score**				
	Dentist-Frankel	3.39 ± 0.50	3.30 ± 0.59	1.166	0.249
	Parents-Venham	0.72 ± 0.55	0.80 ± 0.59	1.531	0.132
	Children-MCDAS	14.93 ± 4.12	14.52 ± 3.93	1.236	0.222
	SCO concentration	7.44 ± 2.43	7.59 ± 2.42	1.563	0.124

*APT, anti-anxiety auricular-plaster therapy; CDA, child dental anxiety; Control, auricular-plaster therapy without anti-anxiety effects; MCDAS, modified child dental anxiety scale in Chinese; SCO, salivary cortisol; TSD, tell-show-do*.

*p < 0.05 for comparisons of TSD + APT versus TSD + control.

Notably, the difference in children's heart rates between the two methods (anti-anxiety method vs. control auricular plasters) was statistically significant at all three treatment points (waiting, anesthesia, and caries removal stage), but this difference was observed only in the subgroup of 21 children with trait anxiety (*P* < 0.05). These children had statistically significant differences in their Frankl (dentist) and Venham (parent) scores, as well as their MCDAS (self) scores (*P* < 0.05). Additionally, a statistically significant difference in salivary cortisol levels was observed between the two methods in children with trait anxiety, but not in those without trait anxiety (*P* < 0.05).

## Discussion

CDA has a high prevalence, affecting between 10 and 50% of children aged 6–12 (3, 4, 6). This study demonstrated that the average heart rate and SCO concentration of children treated with anti-anxiety auricular plasters were lower than those of children treated with control auricular plasters during the three phases of treatment (Table 1). Similarly, children's obedience was improved, as measured by the treating dentist, the children, and their parents during caries removal stage. Thus, when anti-anxiety APT was combined with TSD, a greater reduction in CDA was observed than when control APT was combined with TSD. Further analysis of the data (Table 2) revealed that when the anti-anxiety APT was used instead of the control APT, the average heart rate and SCO of the children with trait anxiety were significantly lower, whereas there was no significant difference between the methods in the children with non-trait anxiety. Similarly, in the tripartite scoring of obedience, the children with trait anxiety were more obedient when treated with the anti-anxiety APT than with the control APT, with no significant difference observed between the two methods in the children without trait anxiety. These results suggest that the anti-anxiety APT was effective at reducing CDA, particularly in children with trait anxiety.

A previous study established a positive correlation between CDA symptoms and self-perceived stress during dental treatment (26). The human neuroendocrine system is primarily responsible for regulating the stress response (27, 28), including the autonomic nervous system (ANS) and hypothalamic pituitary adrenal (HPA) axis. The sympathetic nervous system (SNS) and the parasympathetic nervous system (PNS) regulate the ANS in a two-way feedback loop (29). When the sympathetic nervous system becomes more active, somatic symptoms such as increased heart rate, elevated blood pressure, muscular tension, and perspiration occur to adapt to the environment. Studies have found that individuals with trait anxiety are more likely to have an overactive SNS (30–32). Anti-anxiety APTs have been shown to regulate the ANS, thereby preventing the heart rate from increasing and alleviating CDA symptoms (33). APT does not appear to have anti-anxiety properties in children with a normally functioning autonomic nervous system, which is consistent with TCM theory.

The HPA axis is another neuroendocrine system that influences the body's stress response (29). Cortisol is the end product of the HPA axis, and salivary cortisol levels reflect changes in serum cortisol (34), which can be used as a biochemical indicator for changes in anxiety and stress (35). Therefore, the salivary cortisol level was used as an indicator for CDA in this study (36). Several studies have demonstrated that individuals with trait anxiety's HPA axis respond more strongly to stress than those without trait anxiety, resulting in greater activation (37). During dental treatment, children with trait anxiety have a lower stress response threshold than children without trait anxiety (38), which increases the excitability of the HPA axis, resulting in a greater rise in cortisol (39). In this study, cortisol levels were reduced in children with trait anxiety following anti-anxiety APT (but not control APT), possibly due to decreased HPA axis activity (14). Cortisol levels were not significantly different in children with non-trait anxiety, possibly due to the relatively stable HPA axis in these children.

There are several limitations to consider when interpreting the study's findings. To begin, this was a small study, with only 75 patients completing both treatment arms (of 80 recruited). As a result of the test's applicability, we recruited only 9- to 10-year-old Han Chinese children with chronic untreated caries for the study. Therefore, it is unknown whether the findings are generalizable to children of other ages or ethnicities. In summary, APT was beneficial to children who had trait anxiety but had no effect on children who did not have trait anxiety. These findings suggest that APT may be able to alleviate CDA in some children by reducing physical tension caused by anxiety, possibly through the regulation of the ANS and HPA axes. We believe that by combining APT and TSD, we can improve the cooperation of children who do not have acute dental diseases, as well as alleviate other diseases associated with trait anxiety. Even with this method, we could significantly reduce the use of sedation and general anesthesia in children with trait anxiety who have an oral problem.

## Data Availability Statement

The original contributions presented in the study are included in the article/supplementary material, further inquiries can be directed to the corresponding author.

## Ethics Statement

The studies involving human participants were reviewed and approved by Chinese Clinical Trial Registry (ChiCTR), First Affiliated Hospital of Xinjiang Medical University. Written informed consent to participate in this study was provided by the participants' legal guardian/next of kin.

## Author Contributions

JW, JZ, and DS contributed to the study's design and execution, as well as to the manuscript's writing and review, and all vouch for its intellectual and technical content. All authors contributed to the article and approved the submitted version.

## Funding

This work was supported by the Natural Science Foundation of Xinjiang Uygur Autonomous Region (2018D01C208), Key projects of Hangzhou health science and technology plan (ZD20220080), and Special science and technology support project for the development of biomedicine and health industry in Hangzhou (2021WJCY298).

## Conflict of Interest

The authors declare that the research was conducted in the absence of any commercial or financial relationships that could be construed as a potential conflict of interest.

## Publisher's Note

All claims expressed in this article are solely those of the authors and do not necessarily represent those of their affiliated organizations, or those of the publisher, the editors and the reviewers. Any product that may be evaluated in this article, or claim that may be made by its manufacturer, is not guaranteed or endorsed by the publisher.
